# Investigating the Effect of Selected Non-*Saccharomyces* Species on Wine Ecosystem Function and Major Volatiles

**DOI:** 10.3389/fbioe.2018.00169

**Published:** 2018-11-13

**Authors:** Bahareh Bagheri, Paolo Zambelli, Ileana Vigentini, Florian Franz Bauer, Mathabatha Evodia Setati

**Affiliations:** ^1^Department of Viticulture and Oenology, Institute for Wine Biotechnology, Stellenbosch University, Stellenbosch, South Africa; ^2^Department of Food, Environmental and Nutritional Sciences, University Degli Studi di Milano, Milan, Italy

**Keywords:** wine fermentation, population dynamics, yeast-yeast interactions, multi-starter fermentation, yeast consortium

## Abstract

Natural alcoholic fermentation is initiated by a diverse population of several non-*Saccharomyces* yeast species. However, most of the species progressively die off, leaving only a few strongly fermentative species, mainly *Saccharomyces cerevisiae*. The relative performance of each yeast species is dependent on its fermentation capacity, initial cell density, ecological interactions as well as tolerance to environmental factors. However, the fundamental rules underlying the working of the wine ecosystem are not fully understood. Here we use variation in cell density as a tool to evaluate the impact of individual non-*Saccharomyces* wine yeast species on fermentation kinetics and population dynamics of a multi-species yeast consortium in synthetic grape juice fermentation. Furthermore, the impact of individual species on aromatic properties of wine was investigated, using Gas Chromatography-Flame Ionization Detector. Fermentation kinetics was affected by the inoculation treatment. The results show that some non-*Saccharomyces* species support or inhibit the growth of other non-*Saccharomyces* species in the multi-species consortium. Overall, the fermentation inoculated with a high cell density of *Starmerella bacillaris* displayed the fastest fermentation kinetics while fermentation inoculated with *Hanseniaspora vineae* showed the slowest kinetics. The production of major volatiles was strongly affected by the treatments, and the aromatic signature could in some cases be linked to specific non-*Saccharomyces* species. In particular, *Wickerhamomyces anomalus* at high cell density contributed to elevated levels of 2-Phenylethan-1-ol whereas *Starm. bacillaris* at high cell density resulted in the high production of 2-methylpropanoic acid and 3-Hydroxybutanone. The data revealed possible direct and indirect influences of individual non-*Saccharomyces* species within a complex consortium, on wine chemical composition.

## Introduction

Yeasts are the key components of the wine fermentation ecosystem and responsible for the conversion of grape sugars to CO_2_, ethanol and a kaleidoscope of volatile and non-volatile compounds (Setati et al., 2012; Alonso-del-Real et al., 2017; Mezzasalma et al., 2018). While the initial species composition of this ecosystem in a freshly pressed grape juice will be specific and unique to each juice, several species are found in most musts, independent of grape variety or region of origin (Sun et al., 2009; Bezzera-Bussoli et al., 2013; Milanović et al., 2013; Tristezza et al., 2013; Vigentini et al., 2016; Alonso-del-Real et al., 2017; Mezzasalma et al., 2017). Such common species can be considered as the core of the wine fermentation ecosystem. These species interact and compete with one another, and species with a higher relative fitness will persist longer and significantly influence the chemical composition and sensorial features of the final product (Tristezza et al., 2013; Wang et al., 2015; García et al., 2017).

The performance of each species is affected by abiotic factors such as the availability of nutrients, pH and oxygen, and biotic factors such as initial cell density and the presence of other species (Sadoudi et al., 2012; Medina et al., 2013; Taillandier et al., 2014; Alonso-del-Real et al., 2017; Shekhawat et al., 2017; González et al., 2018). While the impact of abiotic factors on wine fermentations has been investigated in many studies, relatively limited information regarding the impact of biotic factors is available. In particular, it remains unclear whether species-specific ecological interactions will have predictable consequences on the persistence of species, independently of the environmental conditions within individual grape musts.

Data thus far have shown that species such as *Hanseniaspora uvarum, Starm. bacillaris, Lachancea thermotolerans*, and *Torulaspora delbrueckii*, tend to persist longer throughout spontaneous fermentation when present at high initial cell densities (10^4−6^) in grape must or inoculated at higher cell ratios than *S. cerevisiae* (e.g., 1000:1 and 10000:1) in simultaneous or sequential fermentations (Sun et al., 2009; Comitini et al., 2011; Gobbi et al., 2013; Medina et al., 2013; Bagheri et al., 2015; Liu et al., 2016; García et al., 2017). In contrast, the presence of some non-*Saccharomyces* species such as *Metchnikowia pulcherrima* and *Williopsis saturnus* at high cell densities only has a marginal effect on their competitiveness and persistence throughout fermentation (Moreira et al., 2005; Lee et al., 2013). Follow-up studies of several combinations of mixed-culture fermentations using one non-*Saccharomyces* species and *S. cerevisiae* showed that inoculation of some non-*Saccharomyces* species such as *Hanseniaspora guilliermondii, H. vineae, M. pulcherrima, W. anomalus*, and *Starm. bacillaris* at high cell densities generated wines with a distinctive aromatic profile, different from those produced by single cultures of *S. cerevisiae* (Gobbi et al., 2013; Medina et al., 2013; Izquierdo Cañas et al., 2014; Sun et al., 2014; González et al., 2018; Nisiotou et al., 2018). This can either be due to simple addition of metabolites produced by each yeast or specific metabolic activities that may be induced by the presence of other species (Gobbi et al., 2013; Ciani and Comitini, 2015). For instance, wines generated by fermentations with *H. vineae* and *S. cerevisiae* were characterized by higher concentrations of 2-phenylethyl acetate whereas those of *L. thermotolerans* and *S. cerevisiae* produced wines with higher concentrations of 2-phenylethanol (2-Phenylethan-1-ol) and glycerol (Gobbi et al., 2013; Medina et al., 2013). These particular contributions are related to the persistence and competitiveness of the non-*Saccharomyces* strains (Gobbi et al., 2013; Ciani and Comitini, 2015). On the other hand, some non-*Saccharomyces* species have been shown to contribute to wine aromatic profile, despite their lack of persistence in fermentation, which suggests that the contribution to aroma compounds is due to specific enzymatic activities such as β-glucosidase and esterase (Viana et al., 2011; Ciani and Comitini, 2015; De Ovalle et al., 2018). For instance, Sauvignon blanc fermentations inoculated with *S. cerevisiae* and *M. pulcherrima* were reported to generate wines with increased levels of ethyl 2-phenylacetate, 3-methylbutyl acetate and terpenols such as hotrienol, terpiniol, and linalool, despite the early rapid decline of *M. pulcherrima* (Sadoudi et al., 2012). Undoubtedly, each species interacts differently with *S. cerevisiae* and with other yeast species present in a wine ecosystem and such interactions influence the dominance and persistence of the yeasts as well as the analytical profiles of the wines (Gobbi et al., 2013; Medina et al., 2013; Ma et al., 2017). Indeed, some studies have reported antagonistic interactions between *S. cerevisiae* and *L. thermotolerans* (Gobbi et al., 2013), *Starm. bacillaris* (Sun et al., 2014), *T. delbrueckii* (Taillandier et al., 2014), *H. guilliermondii* (Albergaria et al., 2010), *Starm. bacillaris* (Englezos et al., 2016a, 2018a), and *Brettanomyces bruxellensis* (Branco et al., 2014). Such interactions have been attributed to cell-cell contact (Gobbi et al., 2013; Englezos et al., 2016a), restricted oxygen availability (Shekhawat et al., 2017; Englezos et al., 2018a), and production of antimicrobial peptides by *S. cerevisiae* (Branco et al., 2017). Conversely, other studies have demonstrated positive metabolic interaction between *S. cerevisiae* and *Pichia fermentans* (Ma et al., 2017). However, the results indicate that these interactions are strain-specific.

Taken together, most of the previous studies have focused on two-species mixed-culture fermentations (Medina et al., 2013; Taillandier et al., 2014; Branco et al., 2017; De Ovalle et al., 2018). Thus, we have a limited understanding of the effect of individual species in a multi-species yeast community. Furthermore, no information is available on potential interactions among non-*Saccharomyces* species and how such interactions may affect wine ecosystem and wine aroma. Previously, we reported on the establishment of a multi-species yeast consortium that proved to be a suitable approximation of a grape must ecosystem (Bagheri et al., 2017). The population dynamics of the multi-species consortium was evaluated by viable counts and Automated Ribosomal Intergenic Spacer Analysis (ARISA) methods. Similar to other methods, ARISA can introduce bias since it is unable to differentiate between live and dead cells. However, our data confirmed that ARISA and viable counts generated similar growth patterns for individual yeast species in the consortium throughout the fermentation. Furthermore, the population dynamics of the consortium closely resembled that of natural fermentations, making it a suitable model to assess yeast-yeast interactions in complex communities (Bagheri et al., 2017). The current study sought to understand the effect of individual yeast species on a wine yeast consortium. For this purpose, variation in cell density was used as a tool to understand how presence of individual species affects fermentation kinetics, population dynamics of the yeast consortium and the analytical profile of wines.

## Materials and methods

### Yeast consortium and culture conditions

A yeast consortium comprising seven yeast strains obtained from the culture collection of the Institute for Wine Biotechnology (IWBT) and a commercial yeast *S. cerevisiae* Lalvin EC1118 (Lallemand, Canada) were constructed as described in Bagheri et al. (2017). The yeast species used in the constructed yeast consortium are presented in Table 1. The yeast stock cultures were maintained in 20% (v/v) glycerol at −80°C and were streaked out on Wallerstein Laboratory Nutrient agar (WLN) (Sigma-Aldrich, Spain), when required. The plates were incubated at 30°C for 3–5 days.

**Table 1:** Strains used in the yeast consortium.

**Strains**	**Strains codes**	**Strains number**
*Metschnikowia pulcherrima*	*Mp*	Y981
*Candida parapsilosis*	*Cp*	Y842
*Pichia terricola*	*Pt*	Y974
*Wickerhamomyces anomalus*	*Wa*	Y934
*Hanseniaspora vineae*	*Hv*	Y980
*Lachancea thermotolerans*	*Lt*	Y973
*Starmerella bacillaris*	*Sb*	Y975
*Saccharomyces cerevisiae*	*Sc*	EC1118

*The strain codes are an abbreviation of the name of each strain*.

### Microfermentations

Fermentations were performed in synthetic grape juice medium (SGJM) at pH 3.5 (adapted from Henschke and Jiranek (1993) and Bely et al. (1990). The medium contained 200 g/L sugars (100 g/L glucose and 100 g/L fructose) and 300 mg/L assimilable nitrogen (460 mg/L NH_4_Cl and 180 mg/L amino acids). SGJM comprises major constituents including carbon, nitrogen, vitamins, minerals, organic acids and anaerobic factors necessary for yeast growth, formulated to closely mimic natural grape juice. However, lacks polyphenols, grape proteins, varietal thiols, and terpenes that provide the precursors to allow determination of yeast contribution to varietal aroma. Nonetheless, this medium provides a simple matrix in which reproducible data on yeast fermentation performance, gene expression patterns, and microbial interactions can be generated (Riou et al., 1997; Viana et al., 2014). Such reproducibility cannot be achieved in natural grape juice which can vary considerably between vintages and varietals.

**Figure 1:**
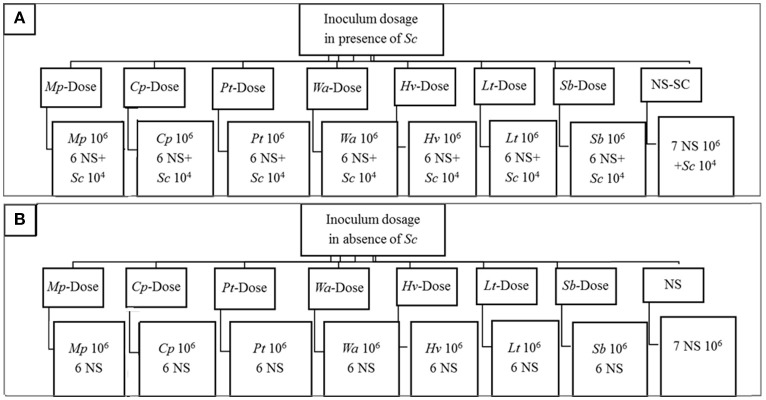
Outline of the experimental plan showing the dosage treatments with *S. cerevisiae* included in the inoculum **(A)** and in the absence of *S. cerevisiae* **(B)** Treatments are defined based on the strain codes. The following abbreviations were used in this figure. *Mp, M. pulcherrima; Cp, C. parapsilosis; Pt, P. terricola; Wa, W. anomalus; Hv, H. vineae; Lt, L. thermotolerans; Sb, Starm. Bacillaris;* NS, Non-*Saccharomyces species*. In the presence of *S. cerevisiae*, in each treatment, one non-*Saccharomyces* species was inoculated at approximately 10^6^ while the rest of yeast species in the consortium (7 NS and Sc) was inoculated at approximately 10^4^. In the absence of *S. cerevisiae*, the same inoculation strategy was applied. However, *S. cerevisiae* was not included in the yeast consortium. In control fermentations, in the presence of *S. cerevisiae* (NS-SC consortium), each NS species was inoculated at approximately 10^6^ and *Sc* at approximately 10^4^ whereas in the absence of *Sc* (NS consortium), 7 NS was each inoculated at approximately 10^6^.

The effect of cell density on the dynamics of yeast consortium was evaluated in presence and in absence of *S. cerevisiae* (Figures 1A,B). In the presence of *S. cerevisiae*, in each treatment, one non-*Saccharomyces* species was inoculated at approximately 10^6^ cells/mL while the rest of yeast species in the consortium (6 non-*Saccharomyces* species and *S. cerevisiae*) was inoculated at approximately 10^4^ cells/mL. In the absence of *S. cerevisiae*, the same inoculation strategy was applied. However, *S. cerevisiae* was not included in the yeast consortium. The inoculation treatments are labeled as X-dose, where (X) represents the respective yeast species that is inoculated at higher concentration. The control fermentations were performed in the presence of *S. cerevisiae* (NS-SC consortium) where each non-*Saccharomyces* species was inoculated at approximately 10^6^ cells/mL and *Sc* at approximately 10^4^ cells/mL. In contrast, in the absence of *S. cerevisiae* (NS consortium), 7 non-*Saccharomyces* was each inoculated at approximately 10^6^ cells/mL.

The fermentations were performed in 500 mL synthetic grape juice dispensed in 500 mL Erlenmeyer flasks fitted with fermentation locks. Static fermentations were carried out in triplicate, at 25°C. The samples were withdrawn at 2-day intervals to monitor the fermentation kinetics. Glucose and fructose were measured, using enzymatic kits, Enzytec™Fluid D-glucose (E5140), fructose (E5120) (Boehringer Mannheim, R-biopharm, Darmstadt, Germany). The fermentations were considered finished when the residual sugar (glucose and fructose) was less than 2 g/L.

### ARISA

Previously, we have proved that ARISA and viable counts generated similar growth patterns for individual yeast species in the yeast consortium in wine fermentation (Bagheri et al., 2017). Thus, in the current study, ARISA was used to monitor the population dynamics throughout the fermentations. For this purpose, two-milliliter samples were withdrawn from fermentation flasks. Samples were centrifuged at 5630 × *g* for 10 min to collect the cells. Genomic DNA was extracted using the method described by Sambrook and Russell (2006). Concentrations of DNA samples were determined spectrophotometrically using the NanoDrop®ND-1000 (NanoDrop Technologies Inc., Wilmington, DE, USA). The concentration of all DNA samples was adjusted to 100 ng/μL. ITS1-5.8S rRNA-ITS2 gene was amplified using the carboxy-fluorescein labeled ITS1 primer (5′-6-FAM- TCC GTA GGT GAA CCT TGC GG-3′) and ITS4 (5′- TCC GTA GGT GAA CCTTGC GG-3′) as described in Slabbert et al. (2010).

ARISA fragments were separated by capillary electrophoresis on an ABI 3010x Genetic Analyser (Applied Biosystems) with a ROX 1.1 labeled size standard (75–1121 base pairs). ARISA profiles were analyzed using Genemapper software version 4.1 (Applied Biosystems). Only fragments with peak area larger than 0.5% of the total fluorescence were considered for further analysis. A bin size of 3 bp for species with ITS region below 700 bp and 5 bp for species with ITS region above 700 bp, was used to minimize the inaccuracies in the ARISA analysis (Slabbert et al., 2010). The abundance of each peak was calculated, dividing individual peak area by the total peak areas for the respective sample.

### Analytical methods

The volatile compounds of wines from different treatments were analyzed by liquid-liquid extraction method, using GC-FID as described by Louw et al. (2010). In brief, the extraction was performed by the addition of 4-methyl-2-pentanol as the internal standard (final concentration 5 mg/L) and 1 mL diethyl ether to each sample. The samples were sonicated for 5 min followed by centrifugation at 4000 × *g* for 5 min. The ether layer (supernatant) was removed and dried over Na_2_SO_4_. Separation of compounds was done, using a DB-FFAP capillary column (Agilent, Little Falls, Wilmington, USA) with dimensions 60 m length × 0.32 mm i.d. × 0.5 μm film thickness. Furthermore, a Hewlett Packard 6890 Plus GC instrument (Little Falls, USA) equipped with a split/splitless injector and a flame ionization detector (FID) were used for gas chromatography (GC). The gas chromatography was performed under the following conditions: an initial oven temperature of 33°C for 17 min, followed by an increase in temperature up to 240°C, for 5 min (12°C/min). Finally, three microliters of the diethyl-ether extract were injected at 200°C in split mode, with the split ratio of 15:1 and the split flow rate of 49.5 mL/min. The column flow rate was 3.3 mL/min, using hydrogen as carrier gas. The detector temperature was 250°C.

### Statistical analysis

All the fermentations and the chemical analysis were performed in at least three repeats. The values were presented as means ± SD. The differences between treatments were determined using analysis of variance (ANOVA) using the statistical software, Statistica version 13.0 (StatSoft Inc., Tulsa, Oklahoma, USA). The differences were considered significant should the *p*-values be equal to or less than 0.05. For multivariate data analysis, the principal component analysis was performed, using XLSTAT in Microsoft® Excel (2016).

## Results

### Fermentation kinetics

The effect of individual yeast species on the wine yeast consortium was evaluated by conducting fermentations in which one of the 8 species was inoculated at a dosage 100 times higher than the rest of the species.

**Figure 2:**
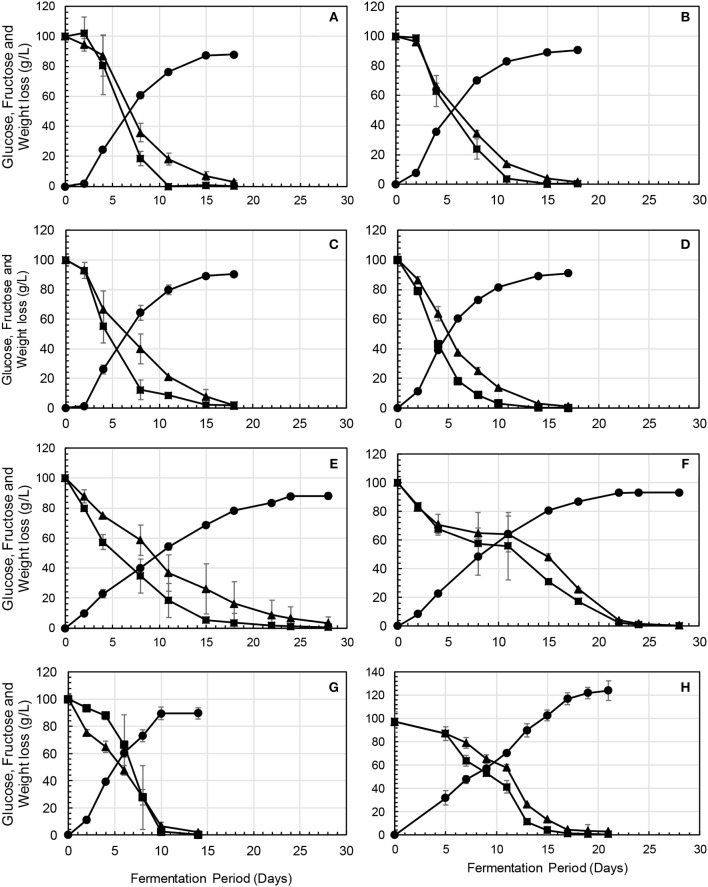
Fermentation profiles showing the kinetics of sugar consumption fructose (▴) and glucose (■), in **(A)** *Mp*-dose, **(B)** *Cp*-dose, **(C)** *Pt*-dose, **(D)**, *Wa*-dose, **(E)** *Hv*-dose, **(F)** *Lt*-dose, **(G)** *Sb*-dose, and **(H)** NS-SC.

Overall, fermentations conducted in the absence of *S. cerevisiae* were sluggish and did not ferment to dryness. Residual sugar ranging between 88 and 107 g/L was detected after 28–32 days when the fermentations were terminated (data not shown). In contrast, all the fermentations in which *S. cerevisiae* was included in the consortium fermented to dryness, albeit at different rates. The fermentations inoculated with a high dosage of *M. pulcherrima, C. parapsilosis*, and *P. terricola*, displayed similar fermentation kinetics, taking 18 days to reach dryness (Figures 2A–C). *H. vineae* and *L. thermotolerans* fermentations displayed the slowest fermentation kinetics, taking 28 and 22 days to reach dryness (Figures 2E,F). In contrast, the fermentation inoculated with a higher concentration of *W. anomalus* and *Starm. bacillaris* (Figures 2D,G) displayed the fastest fermentation kinetics (14 and 12 days) compared to the rest of dosage treatments and the NS-SC control (Figure 2H). The *Starm. bacillaris* high inoculum fermentation displayed rapid consumption of fructose from the onset of fermentation whereas the rest of the treatments showed a similar consumption rate for both glucose and fructose in the early stages of fermentations and a faster consumption of glucose toward the middle of fermentations. Based on the fermentations slope calculated for the first 8 days of fermentations, the *P. terricola* and *W. anomalus* high inoculum fermentations exhibited the fastest glucose consumption.

### Population dynamics

The growth of all species was evaluated during three stages of fermentation, beginning (10–20% sugar consumption), middle (40–60% consumption), and end of fermentation (over 95% sugar consumption).

**Figure 3:**
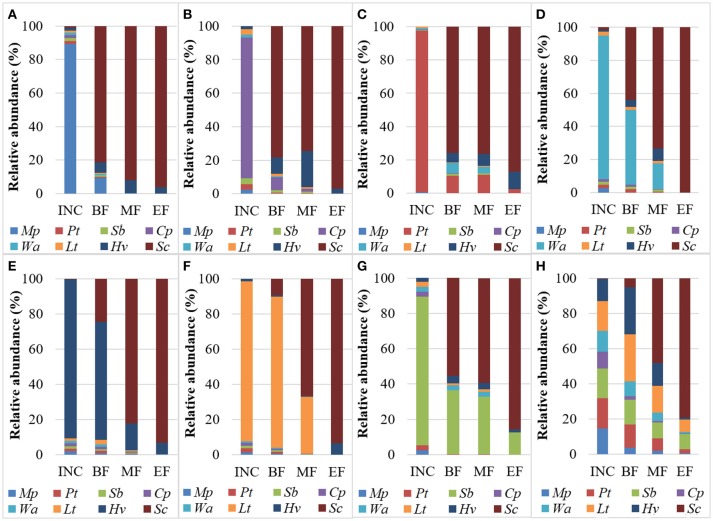
Distribution of yeast species (%) during fermentations at the inoculation time (INC), the beginning (BF), middle (MF), and end of fermentation (EF) in **(A)** *Mp*-dose, **(B)** *Cp*-dose, **(C)** *Pt*-dose, **(D)**, *Wa*-dose, **(E)** *Hv*-dose, **(F)** *Lt*-dose, **(G)** *Sb*-dose, and **(H)** NS-SC.

Overall, a higher initial density of each species allowed the individual species to persist at a slightly higher relative abundance compared to the NS-SC fermentation where all species were inoculated at equal concentrations. However, the dynamics of the individual species were affected differently with regard to their length of persistence. For instance, *M. pulcherrima* and *C. parapsilosis* accounted for less than 10% of the population at the beginning of fermentation and were undetectable by the middle of fermentation (Figure 3). In contrast, *W. anomalus* and *L. thermotolerans* persisted until the middle of fermentation, while *P. terricola, H. vineae*, and *Starm. bacillaris* persisted until the end of fermentation in the respective fermentations where they were inoculated at higher dosages. *Starm. bacillaris* was the only species that persisted until the end of fermentation at a considerable level (12.7%). A high inoculation of some non-*Saccharomyces* species such as *P. terricola* and *C. parapsilosis* supported or inhibited the growth of other non-*Saccharomyces* species in the consortium. For instance, *H. vineae* was detected at higher relative abundance by the middle of fermentation in the *Cp*-dose and *Pt*-dose. Similarly, *W. anomalus* persisted until the middle of fermentation in the *Pt*-dose while it was below detection by the beginning of *Lt*-dose fermentation.

### Production of major volatiles

Production of major volatiles was evaluated for all the wines, in order to reveal a potential contribution of individual non-*Saccharomyces* species in the production of specific major volatiles. The NS-SC produced wine with the lowest total esters (6.52 mg/L) and volatiles acids (9.93 mg/L). *Mp*-dose, *Cp*-dose, and *Pt*-dose wines contained the lowest levels of higher alcohols while they generated the highest concentration of ethyl octanoate, ethyl decanoate, octanoic acid, and decanoic acid (Table 2). In contrast, the *Hv*-dose generated the highest total acetates (117.36 mg/L) and volatile acid (16.06 mg/L), which could be attributed to high ethyl ethanoate and hexanoic acid, respectively. Furthermore, the *Hv*-dose produced 1105 mg/L ethanoic acid, which was the highest amongst all the inoculation regimes. Conversely, the highest concentration of 3-Hydroxybutanone (15.89 mg/L) was recorded in the *Sb*-dose derived wine whereas the highest concentration of 2-Phenylethan-1-ol (25.81 mg/L) was generated in *Wa*-dose wine.

**Table 2:** Major volatile compounds of wines produced from different treatments.

**Compound**	***Mp*-dose**	***Cp*-dose**	***Pt*-dose**	***Wa*-dose**	***Hv*-dose**	***Lt*-dose**	***Sb*-dose**	**NS-SC**
**ETHYL ESTERS**								
Ethyl octanoate	0.33 ± 0.01^d^	0.40 ± 0.05^e^	0.34 ± 0.04^d^	0.17 ± 0.02^bc^	0.1 ± 0^a^	0.14 ± 0.01^ab^	0.09 ± 0.01^a^	0.22 ± 0.02^c^
Ethyl decanoate	0.59 ± 0.04^d^	0.93 ± 0.30^e^	0.71 ± 0.21^d^	0.28 ± 0.04^bc^	0.23 ± 0.02^ab^	0.50 ± 0.05^cd^	0.12 ± 0.01^ab^	0.02 ± 0^a^
Ethyl 2-hydroxypropanoate	8.98 ± 0.23^b^	9.14 ± 0.08^bc^	9.08 ± 0.11^b^	9.50 ± 0.07^cd^	9.31 ± 0.22^bc^	9.73 ± 0.27^de^	10.05 ± 0.09^e^	5.60 ± 0.06^a^
Diethyl butanedioate	0.00^a^	0.00^a^	0.00^a^	0.00^a^	0.00^a^	0.00^a^	0.00^a^	0.68 ± 0.13^b^
∑ Ethyl esters	9.9 ± 0.28	10.47 ± 0.43	10.13 ± 0.36	9.741 ± 0.13	9.64 ± 0.24	10.37 ± 0.33	10.26 ± 0.11	6.52 ± 0.21
**ACETATE ESTERS**								
Ethyl ethanoate	21.97 ± 7.1^a^	39.48 ± 2.3^b^	23.8 ± 0.47^a^	43.85 ± 1.45^b^	114.73 ± 8.7^d^	45.19 ± 2.8^b^	38.1 ± 1.16^b^	82.71 ± 4.5^c^
Ethyl 2-phenylacetate	1.46 ± 0.04^c^	1.74 ± 0.12^c^	1.61 ± 0.6^c^	1.37 ± 0.08^b^	1.33 ± 0.02^b^	1.34 ± 0.01^b^	1 ± 0.02 ^a^	1.02 ± 0.1^a^
2-Phenylethyl acetate	1.67 ± 0.01^d^	1.41 ± 0.01^bc^	1.55 ± 0.01^cd^	1.3 ± 0.01^b^	0.89 ± 0.03^a^	0.86 ± 0.01^a^	0.79 ± 0.03^a^	0.84 ± 0.03^a^
3-Methylbutyl acetate	0.32 ± 0.01^a^	0.38 ± 0.03^abc^	0.32 ± 0.01^a^	0.45 ± 0.01^cd^	0.41 ± 0.08^bcd^	0.48 ± 0.01^d^	0.35 ± 0.03^ab^	0.69 ± 0.04^e^
∑ Acetates	25.42 ± 7.15	43.01 ± 2.46	27.28 ± 1.09	46.97 ± 1.55	117.36 ± 8.83	47.87 ± 2.83	40.24 ± 1.24	85.26 ± 4.67
**ALCOHOLS**								
3-Methylbutan-1-ol	67.64 ± 1.8^a^	79.95 ± 2.3^bc^	74.12 ± 3.8^ab^	102.26 ± 1^de^	84.44 ± 1.57^c^	106.07 ± 4.9^e^	96.6 ± 5.38^d^	100.39 ± 4^de^
2-Phenylethan-1-ol	22.01 ± 1.2^bc^	21.33 ± 2.11^b^	21.31 ± 1.7^ab^	25.81 ± 2.41^e^	18.72 ± 1.9^a^	23.40 ± 2.2^cd^	24.53 ± 3.1^cd^	22.56 ± 3.1^bc^
2-Methylpropan-1-ol	19.39 ± 0.75^b^	18.95 ± 2.2^b^	20.1 ± 0.5^bc^	28.33 ± 1.84^d^	18.3 ± 1.41^ab^	22.6 ± 0.67^c^	26.71 ± 0.73^d^	15.96 ± 0.31^a^
Butan-1-ol	0.52 ± 0.01^a^	0.58 ± 0.03^ab^	0.55 ± 0.04^a^	0.87 ± 0.14^c^	0.81 ± 0.14b^c^	0.96 ± 0.1^c^	0.93 ± 0.08^c^	0.88 ± 0.09^c^
Propan-1-ol	15.18 ± 3.6^a^	30.05 ± 4.4^bc^	21.55 ± 4.1^ab^	35.6 ± 3.34^cd^	43.32 ± 7.2^de^	47.28 ± 6.5^de^	31.16 ± 0.2^bc^	46.52 ± 6.03^e^
3-Ethoxypropan-1-ol	4.73 ± 0.34^b^	5.48 ± 0.96^bc^	5.97 ± 0.56^c^	6.33 ± 0.31^c^	3.04 ± 0.26^a^	2.59 ± 0.23^a^	3.29 ± 0.21^a^	2.65 ± 0.27^a^
∑ Higher alcohols	129.47 ± 7.7	156.34 ± 12	143.6 ± 10.7	199.2 ± 9.04	168.63 ± 13.48	202.9 ± 14.6	183.22 ± 9.7	188.96 ± 13.8
**VOLATILE ACIDS**								
Ethanoic acid	837.3 ± 1.8^d^	853.24 ± 2.5^d^	881.8 ± 3.15^d^	719.9 ± 51^bc^	1105 ± 1.87^e^	639.9 ± 2^ab^	742.4 ± 6.7^c^	614.36 ± 8.9^a^
Propanoic acid	2.15 ± 0.06^b^	3.68 ± 0.60^def^	2.61 ± 0.17^c^	3.77 ± 0.02^ef^	3.28 ± 0.17^d^	3.47 ± 0.2^de^	4.07 ± 0.21^f^	0.96 ± 0.04^a^
2-Methylpropanoic acid	1.07 ± 0.03^ab^	1.19 ± 0.02^bc^	1.18 ± 0.04^bc^	1.35 ± 0.14^cd^	1.65 ± 0.21^f^	1.3 ± 0.05^cd^	1.44 ± 0.03^e^	0.89 ± 0.02^a^
Butanoic acid	0.85 ± 0.03^c^	0.82 ± 0.04^c^	0.85 ± 0.06^c^	0.77 ± 0.01^bc^	0.71 ± 0.02^ab^	0.65 ± 0^a^	0.74 ± 0^ab^	2.27 ± 0.06^d^
3-Methylbutanoic acid	0.85 ± 0.01^ab^	0.89 ± 0.04^ab^	0.88 ± 0.04^ab^	1.06 ± 0.03^bc^	0.91 ± 0.04^ab^	0.96 ± 0.01^abc^	1.15 ± 0.01^c^	0.84 ± 0.19^a^
Pentanoic acid	0.81 ± 0.04^bc^	1.78 ± 0.06^e^	0.93 ± 0.14^c^	1.09 ± 0^d^	0.65 ± 0.06^a^	0.74 ± 0.07^ab^	0.79 ± 0.02^abc^	1.11 ± 0.03^d^
Hexanoic acid	0.44 ± 0.01^a^	1.13 ± 0.03^b^	0.42 ± 0.02^a^	0.4 ± 0.02^a^	5.72 ± 0.19^c^	0.31 ± 0.08^a^	0.41 ± 0.02^a^	1.20 ± 0.17^b^
Octanoic acid	3.36 ± 0.02^d^	3.28 ± 0.01^d^	3.45 ± 0.08^d^	2.32 ± 0.09^c^	1.39 ± 0.8^ab^	1.37 ± 1.01^ab^	1.2 ± 0.04^a^	1.70 ± 0.01^b^
Decanoic acid	2.38 ± 0.04^d^	2.55 ± 0.05^d^	2.72 ± 0.08^d^	1.47 ± 0.01^b^	1.75 ± 0.09^b^	1.85 ± 0.01^bc^	0.83 ± 0.01^a^	0.96 ± 0.01^a^
∑ Volatile acids without ethanoic acid	11.91 ± 2.04	15.32 ± 3.31	13.04 ± 0.63	12.23 ± 0.32	16.06 ± 1.58	10.65 ± 3.23	10.63 ± 0.32	9.93 ± 0.53
**ALDEHYDES AND KETONES**								
3-Hydroxybutanone	7.88 ± 1.3^b^	5.43 ± 0.57^ab^	7.98 ± 0.42^b^	5.23 ± 0.09^ab^	5.44 ± 0.45^ab^	5.28 ± 0.51^ab^	15.89 ± 3.57^c^	2.07 ± 1.07^a^

*Values are represented in mg/L ± standard deviations. Values with different letters in the same row are significantly different (p < 0.05) according to the Tukey test*.

**Figure 4:**
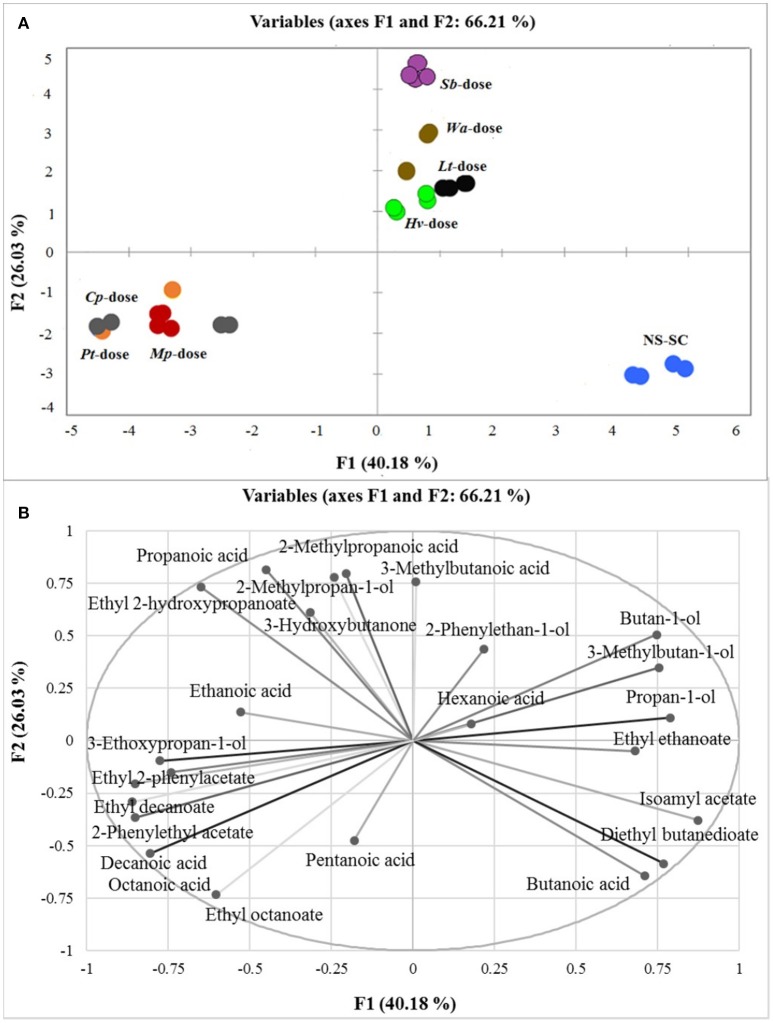
PCA score plot **(A)** and corresponding loading plot **(B)** of first and second principal components (PC) derived from PC analysis of the major volatile compounds produced in synthetic must fermentations. The major volatile compounds are represented by light grays whereas the treatments are as follows: *Mp*-dose (red), *Cp*-dose (gray), *Pt*-dose (orange), *Wa*-dose (brown), *Hv*-dose (green), *Lt*-dose (black), *Sb*-dose (purple), and NS-SC (blue). Some replicates were overlapped in each treatment (e.g. *Pt*-dose).

Principal component analysis (PCA) was applied to all quantifiable major volatiles to decipher the compounds which would drive the differentiation between the wines. PC1 and PC2 explained 66.21% of the variance. PC1 accounts for 40.18% of the variance and mainly shows separation of the wines into three major groups based on rapid (*Cp*-dose, *Mp*-dose, and *Pt*-dose), intermediary (*Wa*-dose, *Hv*-dose, *Lt*-dose, and *Sb*-dose) and slow (NS-SC) establishment of *S. cerevisiae* within the ecosystem. The NS-SC derived wine was clearly separated from the rest of treatments in the lower right quadrant of the PC1 and it was mainly associated with butanoic acid, ethyl ethanoate, 3-methylbutyl acetate, and diethyl butanedioate. Conversely, the *Cp*-dose, *Pt*-dose, and *Mp*-dose wines formed one group and were mainly associated with ethyl decanoate, ethyl octanoate, 2-phenylethyl acetate, ethyl 2-phenylacetate as well as octanoic acid and decanoic acid. PC2 accounts for 26.03% of the variance and further separates the *Sb*-dose wine from the intermediary group. Within this group, the *Sb*-dose wine was associated with 3-methylbutanoic acid, 2-methylpropanoic acid, and 2-methylpropan-1-ol. In contrast, the *Wa*-dose, *Hv-*dose, and *Lt*-dose were associated with hexanoic acid, 3-Methylbutan-1-ol, butan-1-ol, and propan-1-ol (Figure 4).

## Discussion

The current study used the variation in cell densities as a tool to understand the influence of individual species on population dynamics in a complex community and on the chemicals responsible for wine aroma. For this purpose, one out of 8 species was inoculated at a dosage 100 times higher than the rest of the species.

The data showed that the persistence of some species, for instance, *L. thermotolerans, Starm. bacillaris, W. anomalus*, and *H. vineae* were enhanced considerably when they were inoculated at high concentration. The persistence of these species until the final stages of fermentation have been reported previously (Viana et al., 2011; Gobbi et al., 2013; Bagheri et al., 2015). These species have some common metabolic traits such as moderate (*Starm. bacillaris, W. anomalus*, and *H. vineae*) and strong fermentative capabilities (*L. thermotolerans*), high tolerance to different levels of ethanol (6–12%) and osmotic pressure (Sabel et al., 2014; Englezos et al., 2016a; Hranilovic et al., 2018; Martin et al., 2018) that can explain their persistence in wine fermentations. Despite their common metabolic traits, the persistence of each species in wine fermentation can be attributed to their species-specific characteristics. For instance, *L. thermotolerans* has shown to tolerate very high concentration of ethanol (12% v/v) and total SO_2_ (>200 mg/L) (Hranilovic et al., 2018; Nally et al., 2018) whereas *W. anomalus* has displayed high tolerance to low pH (Sabel et al., 2014). Persistence of *Starm. bacillaris* throughout fermentation can be attributed to its strong fructophilic characteristics, limited nitrogen requirements and the ability of this species to excrete some branched amino acids (Englezos et al., 2018b).

Despite the similar pattern observed for the persistence of *L. thermotolerans, Starm. bacillaris, W. anomalus*, and *H. vineae* throughout fermentations, the growth dynamics, as well as the kinetics of the respective fermentation processes, were remarkably divergent. Three patterns of fermentation kinetics comprising fast (*Sb*-dose and *Wa*-dose), intermediate (*Cp*-dose, *Pt*-dose, and *Mp-*dose) and slow (*Lt*-dose and *Hv*-dose) were evident. These trends revealed possible yeast-yeast interactions. For instance, inoculation of *Starm. bacillaris* at a high concentration resulted in fast fermentation kinetics which can be attributed to rapid simultaneous utilization of both fructose and glucose. We can, therefore, infer that there is a co-operative interaction between the two dominant yeasts (i.e., *Starm. bacillaris* and *S. cerevisiae*) in this fermentation. Indeed, several studies have shown that most strains of *Starm. bacillaris* are fructophilic while *S. cerevisiae* is known to exhibit preferential consumption of glucose. This co-operative interaction has been observed amongst other strains of these two species (Magyar and Tóth, 2011; Suzzi et al., 2012; Masneuf-Pomarede et al., 2015; Tofalo et al., 2016). These findings suggest that the interaction between the two could be more species-specific rather than strain specific. Despite the positive interaction observed in *Sb*-dose fermentation between these two species, a gradual decrease in the population of *Starm. bacillaris* was observed. This decline in the population of *Starm. bacillaris* can be due to several parameters such as lower fermentative capability of this species compared to *S. cerevisiae* due to several parameters such as cell to cell contact (Englezos et al., 2019), limited oxygen (Englezos et al., 2018b) and nutrient availability as suggested by Englezos et al. (2016b). In the case of *Wa*-dose fermentation, the sugar consumption rate displayed a faster utilization of glucose than fructose, however, this does not seem to affect the implantation of *S. cerevisiae*.

Within the intermediate fermentations, *M. pulcherrima, C. parapsilosis, P. terricola* were rapidly replaced by *S. cerevisiae* at an early stage of fermentations, despite the high initial density. We can infer that the growth of these species may be curtailed by other parameters. For instance, several strains of *M. pulcherrima* were reported to have lower growth rates than *S. cerevisiae* and required significantly higher oxygen input to grow in high sugar environments (Contreras et al., 2014; Shekhawat et al., 2017). Similar correlations for the growth of *C. parapsilosis* and oxygen availability were reported by Oh et al. (1998) and Holland et al. (2014), while the rapid decline in the population of *P. terricola* in *Pt-*dose could be due to weak fermentative capability of this species as previously suggested by Clemente-Jimenez et al. (2005) and Di Maro et al. (2007). Even though the rapid decline in the population of *M. pulcherrima, C. parapsilosis, P. terricola* can be attributed to their low fermentative capability and limited nutrient and oxygen availability, the distribution of yeast species in the consortium is strongly affected by the growth of individual species and the interaction among these species. It was evident in the current study that a high inoculum of *C. parapsilosis* created conducive conditions for enhanced persistence of *H. vineae*, while *P. terricola* promoted the persistence of both *H. vineae* and *W. anomalus*. This could suggest that at high dosage *C. parapsilosis* and *P. terricola* either produce compounds that retard the establishment of the other yeasts or compete strongly for nutrients. However, due to their sensitivity to oxygen limitation and increasing ethanol, the two yeasts still decline rapidly.

Our data revealed potential competition between *H. vineae, L. thermotolerans*, and *S. cerevisiae*. Indeed, the fermentations inoculated with high concentrations of *L. thermotolerans* and/or *H. vineae* were the slowest, probably due to the moderate (*H. vineae*) and strong (*L. thermotolerans*) fermentation capacity of these species accompanied by a delay in the implantation of *S. cerevisiae*. Medina et al. (2013), demonstrated that *H. vineae* competes with *S. cerevisiae* for the consumption of nitrogen and vitamins. This lack of nutrients retards the growth of *S. cerevisiae* while *H. vineae* is a moderate fermenter and cannot ferment to dryness. On the other hand, previous studies have confirmed that *L. thermotolerans* shows a high degree of competitiveness against *S. cerevisiae* which can retard the fermentation kinetics and delay the implantation of *S. cerevisiae*. Decline in the population of *L. thermotolerans* could be due to competition for oxygen between *S. cerevisiae* and *L. thermotolerans* (Shekhawat et al., 2017), a slow metabolic activity of *L. thermotolerans* under anoxic conditions (Nissen and Arneborg, 2003), cell to cell contact (Gobbi et al., 2013) as well as production of anti-microbial peptides by *S. cerevisiae* (Branco et al., 2017).

Concerning the major volatile production, a clear separation was observed between NS-SC and the dosage treatments, which confirmed that the production of major volatiles was strongly affected by the treatments. Further analysis of chemical compounds underlined that aromatic signature of some non-*Saccharomyces* species could be detected in wine where these species were inoculated at a high concentration. Our study for the first time highlighted that presence of *W. anomalus* at a high cell density could result in an elevated level of 2-Phenylethan-1-ol in wine whereas *C. parapsilosis* at a high cell density could generate the wine with high concentrations of valeric acid, ethyl decanoate, and ethyl octanoate. This result suggests that the presence of *C. parapsilosis* and *W. anomalus* at a high cell density in grape must can affect the quality of wine, despite the rapid decline of these species at the early stage of fermentation. Furthermore, the aromatic signature of *H. vineae* (high concentrations of ethyl ethanoate and ethanoic acid) and *L. thermotolerans* (high concentration of 3-Methylbutan-1-ol and low concentration of ethanoic acid) could be detected in wine where these species were inoculated at a high concentration. Similar results have been reported for wines obtained from the mixed culture fermentations of *S. cerevisiae* and *H. vineae* in other grape matrices such as Macabeo, Merlot, and Chardonnay (Viana et al., 2011; Medina et al., 2013; Lleixà et al., 2016). Similarly, the production of 3-Methylbutan-1-ol and ethanoic acid in *Lt*-dose wine followed a similar pattern in wine obtained from the mixed culture fermentation of *L. thermotolerans* and *S. cerevisiae* in pasteurized commercial white grape juice (Gobbi et al., 2013) and Airén grape juice (Benito et al., 2016). On the other hand, our study for the first time demonstrated that presence of *W. anomalus* at a high cell density could result in an elevated level of 2-Phenylethan-1-ol in wine whereas *C. parapsilosis* at a high cell density could generate the wine with high concentrations of valeric acid, ethyl decanoate, and ethyl octanoate. This result suggests that the presence of *C. parapsilosis* and *W. anomalus* at a high cell density in grape must can affect the quality of wine, despite the rapid decline of these species at the early stage of fermentation.

In conclusion, the data confirmed that persistence of non-*Saccharomyces* species throughout fermentation does not necessarily depend on their initial cell density. Our results reveal that the presence of individual non-*Saccharomyces* species will positively or negatively affect the growth of other species in the consortium. Therefore, the growth of non-*Saccharomyces* species in a multi-species community is affected by both the interactions between non-*Saccharomyces* and *Saccharomyces* species as well as the interactions among non-*Saccharomyces* species in the yeast consortium. Consequently, the production of major volatiles and the quality of wine is affected by these yeast-yeast interactions. In our data, we can link the production of certain compounds to the presence of specific non-*Saccharomyces* species, but only for those that were inoculated at a higher cell density. However, the production of these compounds did not necessarily depend on their persistence through the fermentation. Indeed, presence of some species at a high cell density was sufficient to affect the quality of wine. Additional experiments with adjusted relative inoculation cell densities will be required to better evaluate how the strain- or species-specific interactions within this ecosystem impact on fermentation outcomes. The results also highlight the usefulness of a consortium-based approach to better understand the dynamics of multi-species interactions and their impact on wine character. The result of current study is a first step to untangling the interactions within the wine ecosystem. Future work will focus to unravel mechanisms underlying yeast-yeast interactions observed in this study (metabolically and physically) and to evaluate how presence of two or three non-*Saccharomyces* species at high densities in the consortium affect the population dynamics and wine aroma.

## Author contributions

MS and FB conceptualized the study, Supervised the design of experimental layout. BB and PZ performed the experiments and analyzed data. BB wrote first draft of the manuscript. MS, FB, BB, PZ, and IV edited draft manuscript and approved final manuscript.

### Conflict of interest statement

The authors declare that the research was conducted in the absence of any commercial or financial relationships that could be construed as a potential conflict of interest.
