# Corrigendum: Investigating the Effect of Selected Non-*Saccharomyces* Species on Wine Ecosystem Function and Major Volatiles

**DOI:** 10.3389/fbioe.2019.00140

**Published:** 2019-06-12

**Authors:** Bahareh Bagheri, Paolo Zambelli, Ileana Vigentini, Florian Franz Bauer, Mathabatha Evodia Setati

**Affiliations:** ^1^Department of Viticulture and Oenology, Institute for Wine Biotechnology, Stellenbosch University, Stellenbosch, South Africa; ^2^Department of Food, Environmental and Nutritional Sciences, University Degli Studi di Milano, Milan, Italy

**Keywords:** wine fermentation, population dynamics, yeast-yeast interactions, multi-starter fermentation, yeast consortium

In the original article, there was a mistake in Figure 2 as published. The order of the graphs (A–H) is incorrect and does not match the caption nor the in-text citation. The corrected Figure 2 appears below.

**Figure 2:**
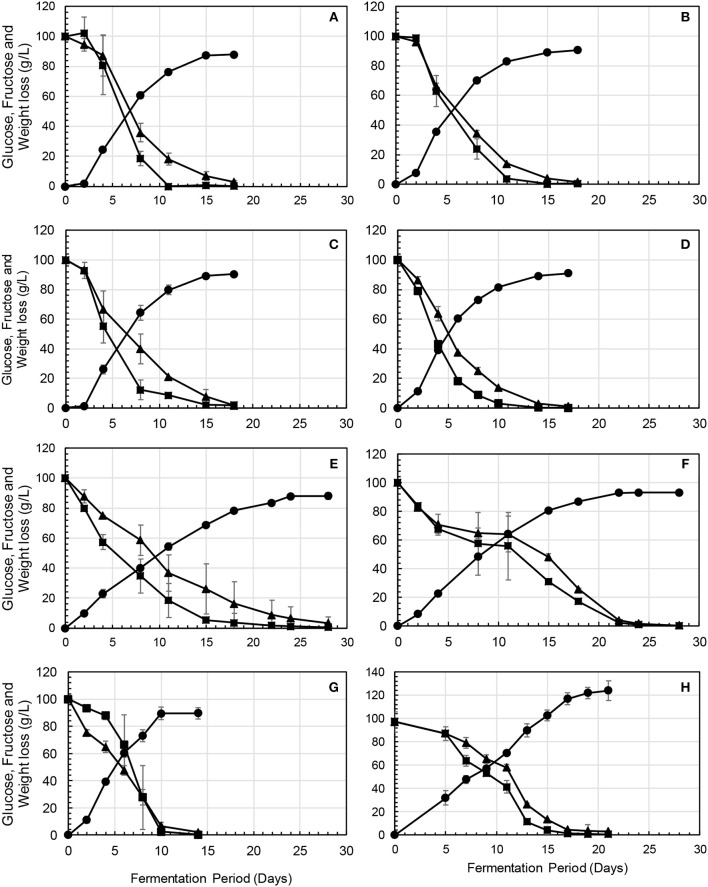
Fermentation profiles showing the kinetics of sugar consumption [fructose (▴) and glucose (■)] and CO_2_ release [weight loss (●)], in **(A)** *Mp*-dose, **(B)** *Cp*-dose, **(C)** *Pt*-dose, **(D)**, *Wa*-dose, **(E)** *Hv*-dose, **(F)** *Lt*-dose, **(G)** *Sb*-dose, and **(H)** NS-SC, in which *Metschnikowia pulcherrima* (*Mp*), *Pichia terricola* (*Pt*), *Wickerhamomyces anomalus* (*Wa*), *Hanseniaspora vineae* (*Hv*), *Lachancea thermotolerans* (*Lt*), and *Starmerella bacillaris* (*Sb*) were inoculated at high levels in the respective treatments, while in the NS-SC treatment they were all inoculated at 10^6^ cfu/mL with *Saccharomyces cerevisiae* (SC) at 10^4^ cfu/mL.

The authors apologize for this error and state that this does not change the scientific conclusions of the article in any way. The original article has been updated.

